# Is concomitant splenectomy beneficial for the long-term survival of patients with gastric cancer undergoing curative gastrectomy? A single-institution study

**DOI:** 10.1186/1477-7819-12-193

**Published:** 2014-06-26

**Authors:** Hao Zhang, Deyan Pang, Huanming Xu, Yuan Ren, Caigang Liu

**Affiliations:** 1 Department of Breast Surgery, Second Hospital of Dalian Medical University, Dalian 116023, China; 2 Department of Mathematics, Northeast Yucai School, Shenyang 110179, China; 3 Department of Transfusion, Fourth Hospital of China Medical University, Shenyang 110032, China; 4 Department of Hematology, First Hospital of China Medical University, Shenyang 110001, China

**Keywords:** Gastric cancer, Splenectomy, Survival

## Abstract

**Background:**

Curative resection is the treatment of choice for gastric cancer, but it is unclear whether gastrectomy should also include splenectomy. We retrospectively analyzed long-term survival in patients in our hospital who underwent gastrectomy plus splenectomy (G + S) or gastrectomy alone (G-A) for gastric cancer.

**Methods:**

We identified 214 patients who underwent surgery with curative intent between 1980 and 2003. Of these, 100 underwent G + S, and 114 underwent G-A. The primary endpoint was 5-year overall survival (OS).

**Results:**

Median follow-up was 18 months in patients who underwent G + S, and 26.5 months in patients who underwent G-A. The 5-year OS rate was significantly higher in patients who underwent G-A (33.8%; 95% CI 24.2 to 43.4%) than in those who underwent G + S (28.8%; 95% CI 19.6 to 38.0%) (log-rank test, *P* = 0.013).

**Conclusions:**

Splenectomy does not benefit patients undergoing gastrectomy for gastric cancer. Routine splenectomy should be abandoned in patients undergoing radical resections for gastric cancer.

## Background

Gastric cancer remains the most common cause of cancer-related deaths worldwide, with poor patient prognosis and a lack of adequate treatment methods. Although surgical resection is the primary treatment method for gastric cancer, more extensive surgery is accompanied by greater risks of surgery-related morbidity and mortality [1]. The mortality rate for patients undergoing gastrectomy surgery in western countries often exceeds 5%, and may be as high as 16% [2], although the rate in Japanese patients has been reported as being less than 2% [3]. Moreover, the efficacy of concomitant splenectomy remains unclear. As our understanding of the role of the spleen in the immunological defenses of the body and in the prevention of sepsis has increased, the value of routine removal of the spleen, *en bloc* with the stomach, during the course of radical, potentially curative resection for gastric cancer has been reassessed.

In this study, we retrospectively analyzed long-term survival data of patients who underwent gastrectomy, with or without splenectomy, for gastric cancer at a single institution. We found that operative complication rates were significantly higher in patients undergoing concomitant splenectomy, and it carried no benefit to patients already undergoing gastrectomy. Consequently, we believe, its routine use in radical resection for gastric cancer should be abandoned.

## Methods

### Patients

We identified 214 patients with histologically confirmed gastric cancer who had undergone radical surgery at the First Affiliated Hospital of China Medical University between 1980 and 2003, Patients were included in the study if they: 1) had histologically confirmed gastric cancer; 2) had undergone curative surgery; 3) had a complete medical record available; and 4) had never received neoadjuvant therapies. The number of patients at every period of diagnosis and the number treated by each surgeon were roughly equal.

All patients were followed up by mail or telephone interviews, and the final follow-up was in December 2008. Clinical, surgical, and pathological findings at the time of surgery and at each follow-up were collected and recorded in the database.

### Ethics approval

The study protocol was approved by the Ethics Committee of China Medical University. [The patients signed the informed consent about operation and so on routinly, we don’t need another consent about this study any more].

### Surgical procedures and classifications of gastric cancer

All operations were performed at the First Affiliated Hospital of China Medical University. Surgical procedures and pathological assessments were standardized in accordance with the Japanese classification of gastric cancer [4]. All patients underwent standard total or distal sub-total gastrectomy, depending on the location and macroscopic appearance of the primary tumor.

### Endpoints and follow-up

The primary endpoint was 5-year overall survival (OS) rate. OS was calculated from the date of surgery until the date of death or final follow-up contact. Patients remaining alive at the date of final follow-up were censored at that point. Patients were followed up every 6 months for the first 5 years after surgery, and every 12 months thereafter.

### Statistical analyses

OS was analyzed in all eligible patients. Survival curves were determined by the Kaplan–Meier method, and compared by the log-rank test. Potential prognostic factors were entered into a Cox regression model. For univariate analyses, prognostic factors of interest and treatment group were deemed covariates in the Cox regression model. Subgroups were analyzed in this model to evaluate interactions between treatment and subgroup. All *P*-values were two-sided, with *P* < 0.05 considered significant. All statistical analyses were performed using SPSS software (version 16.0; SPSS Inc., Chicago, IL, USA).

## Results

Of the 214 patients who underwent gastrectomy for gastric cancer from 1980 to 2003, 100 patients (median age 59 years) underwent gastrectomy plus splenectomy (G + S), while the remaining 114 (median age 55.5 years), underwent gastrectomy alone (G-A). All patients were followed up for at least 5 years, until December 19, 2008.

**Figure 1:**
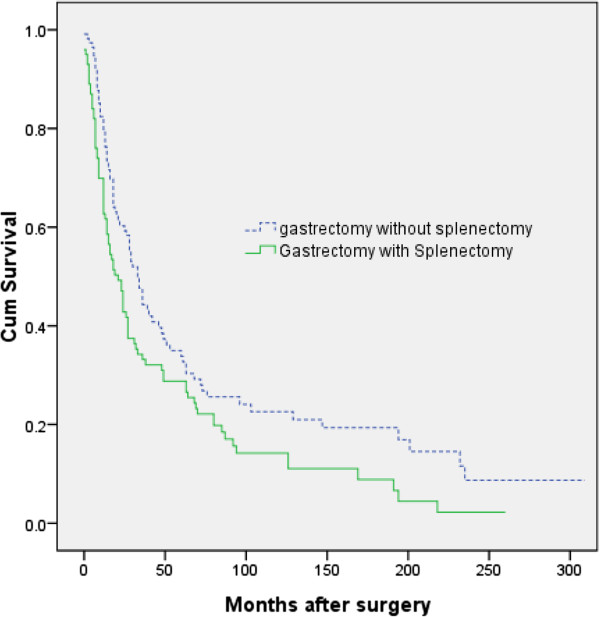
**Kaplan-Meier analysis of overall survival (OS) in patients undergoing gastrectomy plus splenectomy and those undergoing gastrectomy alone.** The 5-year OS rates in these two groups were 28.8% (95% CI 19.6 to 38.0%) and 33.8% (95% CI 24.2 to 43.4%), respectively (*P* = 0.013 by the log-rank test).

The baseline demographic and clinical characteristics of the two groups were similar (Table 1). Of the 214 patients, 13 (6.1%) had early gastric cancer (EGC) confined to the submucosa or mucosa. All patients underwent curative resection. Total gastrectomy was performed in 60 of the 100 (60%) patients who underwent G + S, and in 68 of the 114 (60%) who underwent G-A. Complications were more common (*P* = 0.005) in the G + S group than in the gastrectomy-alone group (Table 1; Table 2). A total of 26 patients, 12% in each group, received adjuvant chemotherapy after surgery.The median follow-up durations were 18 months in the G + S group and 26.5 months in the G-A group. By the end of follow-up, 87 of the 100 patients (87%) who underwent G + S and 83 of the 114 (73%) who underwent G-A had died. Kaplan-Meier analysis of OS in these two groups showed a significant between-group difference (Figure 1). The 5-year OS rates were 33.8% (95% CI 24.2 to 43.4%) in the G-A group and 28.8% (95% CI 19.6 to 38.0%) in the G + S group (p = 0.013 by the log-rank test).

**Table 1:** **Characteristics of patients undergoing gastrectomy with and without splenectomy (n = 214)**^**a**^

**Characteristic**	**G + S (n = 100)**	**G-A (n = 114)**	***P*-value**
Age, years			0.836
≤ 55	32 (32)	38 (33)	
> 55	68 (68)	76 (67)	
Sex			0.190
Men	82 (82)	85 (75)	
Women	18 (18)	29 (25)	
Tumor size, cm			0.393
≤ 4	23 (23)	27 (24)	
5 to 6	26 (26)	21 (18)	
> 6	51 (51)	66 (58)	
Pathological tumor stage			0.600
T1	5 (9)	8 (9)	
T2	28 (49)	45 (53)	
T3	16 (28)	26 (31)	
T4	8 (14)	6 (7)	
Pathological nodal stage			0.153
N0	10 (15)	24 (28)	
N1	20 (31)	29 (34)	
N2	23 (35)	23 (27)	
N3	12 (19)	9 (11)	
TNM stage			0.355
IA	2 (2)	5 (4)	
IB	19 (19)	23 (20)	
II	18 (18)	30 (26)	
IIIA	29 (29)	31 (27)	
IIIB	14 (14)	14 (12)	
IV	18 (18)	11 (10)	
Gross type (Borrman)			0.154
I	3 (3)	2 (2)	
II	20 (20)	12 (11)	
III	59 (60)	66 (61)	
IV	17 (17)	29 (27)	
Type of gastrectomy			0.958
Total	60 (60)	68 (60)	
Sub-total	40 (40)	46 (40)	
Complications	24 (24)	11 (10)	0.005^c^
Adjunctive therapy	12 (12)	14 (12)	0.903

*Abbreviations*: *G + S* gastrectomy plus splenectomy, *G-A* gastrectomy alone, *TMN* tumor, node, metastasis.

^a^Data are given as n (%).

^c^Significant.

**Table 2:** **Complications in patients undergoing gastrectomy with and without splenectomy (n = 214)**^**a**^

**Complications**	**G + S (n = 100)**	**GA (n = 114)**
Intestinal obstruction	1 (1)	3 (3)
Pneumonia	1 (1)	1 (1)
Abdominal abscess	7 (7)	2 (2)
Anastomotic leakage	7 (7)	0 (0)
Other	8 (8)	5 (4)

*Abbreviations*: *G + S* gastrectomy plus splenectomy, *G-A* gastrectomy alone.

^a^Data are given as n (%).

The hazard ratio (HR) for death was 1.456 (95% CI 1.076 to 1.970; *P* = 0.015) in the G + S relative to the G-A group (Table 3). After adjustment for nine baseline variables (age, sex, tumor size, Borrmann type, T stage, lymph-node stage, TNM stage, complications and type of gastrectomy) using Cox regression analysis, the HR was 1.777 (95% CI 1.137 to 2.777; *P* = 0.012) (Table 3). As expected, multivariate analysis showed that Borrmann type IV and advanced TNM stages were significantly associated with poor survival (Table 3).

**Table 3:** HR for death in the intention-to-treat population (n = 214)

	**Univariate analyses**		**Multivariate analyses**	
	**HR (95% CI)**	***P***^**a**^	**HR (95% CI)**	***P***^**b**^
Age, years		0.251		0.242
≤ 55	1 (Ref)		1 (Ref)	
> 55	1.212 (0.872 to 1.685)	0.251	1.427 (0.823 to 2.476)	0.242
Sex		0.444		0.473
Women	1 (Ref)		1 (Ref)	
Men	0.867 (0.600 to 1.251)	0.444	0.756 (0.430 to 1.328)	0.473
Tumor size, cm		0.000		0.172
≤ 4	1 (Ref)		1 (Ref)	
5 to 6	1.492 (0.932 to 2.390)	0.096	1.562 (0.763 to 3.199)	0.222
> 6	2.058 (1.368 to 3.097)	0.001	1.808 (0.928 to 3.522)	0.082
Pathological tumor stage		0.000		0.698
T1	1 (Ref)		1 (Ref)	
T2	4.134 (2.968 to 5.756)	0.000	1.484 (0.828 to 2.658)	0.184
T3	6.508 (4.643 to 9.121)	0.000	1.120 (0.409 to 3.066)	0.826
T4	9.231 (5.752 to 14.813)	0.000	1.763 (0.149 to 3.906)	0.745
Pathological nodal stage		0.000		0.413
N0	1 (Ref)		1 (Ref)	
N1	2.253 (1.912 to 2.654)	0.000	1.201 (0.764 to 1.888)	0.426
N2	4.128 (3.390 to 5.027)	0.000	1.348 (0.586 to 3.100)	0.482
N3	8.215 (6.301 to 10.711)	0.000	1.722 (0.515 to 5.756)	0.377
TNM stage		0.000		0.016^c^
IA	1 (Ref)		1 (Ref)	
IB	2.402 (1.611 to 3.580)	0.000	2.778 (1.504 to 5.132)	0.001^c^
II	4.589 (3.148 to 6.690)	0.000	5.461 (2.049 to 14.559)	0.001^c^
IIIA	8.334 (5.708 to 12.168)	0.000	10.403 (2.567 to 42.162)	0.001^c^
IIIB	11.148 (7.406 to 16.782)	0.000	13.570 (2.182 to 84.410)	0.005^c^
IV	18.123 (9.654 to 28.184)	0.000	27.360 (3.028 to 47.256)	0.003^c^
Gross type (Borrmann)		0.001		0.034^c^
I	1 (Ref)	0.984	1 (Ref)	
II	0.989 (0.343 to 2.853)		3.316 (0.727 to 15.120)	0.122
III	1.337 (0.491 to 3.645)	0.570	3.358 (0.894 to 12.611)	0.073
IV	2.561 (1.909 to 7.210)	0.025	4.584 (1.201 to 17.499)	0.026^c^
Type of gastrectomy		0.463		0.297
Total	1 (Ref)		1 (Ref)	
Sub-total	0.891 (0.654 to 1.213)	0.463	1.139 (0.722 to 1.796)	0.297
Operation		0.015		0.012^c^
Without splenectomy	1 (Ref)		1 (Ref)	
With splenectomy	1.456 (1.076 to 1.970)	0.015	1.777 (1.137 to 2.777)	0.012^c^
Complications		0.887		0.173
No	1 (Ref)		1 (Ref)	
Yes	0.992 (0.894 to 1.101)	0.887	0.502 (0.254 to 1.193)	0.173

*Abbreviations*: *HR* hazard ratio, *Ref* reference category.

^a^Derived from tests of HR for prognostic factors in univariate model adjusted for type of surgery in a Cox proportional-hazards model.

^b^Cox-regression analysis, controlling for the prognostic factors listed in the table.

^c^Significant.

G-A was significantly more beneficial than G + S in men, and in patients with tumor size >6 cm, Borrmann type III tumors, or those who underwent subt-otal gastrectomy (Table 4). We therefore would not recommend routine splenectomy in patients with gastric cancer who require sub-total gastrectomy, unless the tumor lies close to or directly invades the splenic hilum. Our results indicate that splenectomy should not be included in the treatment of patients with curable gastric cancer.

**Table 4:** Tests for heterogeneity of treatment effect according to the clinicopathological characteristics of the patients undergoing gastrectomy with and without splenectomy

**Subgroup**	**G + S, patients/total patients, n**	**G-A, patients/total patients, n**	**HR (95% CI)**^**a**^	***P*-value**^**b**^
Deaths	87/100	83/114	1.456 (1.076 to 1.970)	
Age, years				
≤ 55	26/32	25/38	1.559 (0.897 to 2.709)	0.116
> 55	61/68	58/76	1.377 (0.960 to 1.974)	0.082
Sex				
Women	17/18	20/29	1.527 (0.797 to 2.926)	0.201
Men	70/82	63/85	1.433 (1.018 to 2.018)	0.039^c^
Tumor size, cm				
≤ 4	16/23	15/27	1.606 (0.778 to 3.314)	0.200
5 to 6	23/26	18/21	1.135 (0.604 to 2.134)	0.694
> 6	48/51	50/66	1.606 (1.079 to 2.391)	0.019^c^
Pathological tumor stage				
T1	5/5	7/8	3.458 (0.790 to 15.144)	0.100
T2	21/28	30/45	1.478 (0.840 to 2.598)	0.175
T3	14/16	19/26	0.871 (0.426 to 1.781)	0.706
T4	7/8	4/6	1.803 (0.521 to 6.238)	0.352
Pathological nodal stage				
N0	7/10	15/24	1.800 (0.707 to 4.586)	0.218
N1	16/20	19/29	1.373 (0.704 to 2.678)	0.353
N2	21/23	19/23	1.221 (0.648 to 2.300)	0.537
N3	11/12	7/9	0.969 (0.345 to 2.722)	0.952
TNM stage				
IA	2/2	5/5	2.783 (0.386 to 20.066)	0.310
IB	16/19	14/23	1.433 (0.694 to 2.961)	0.331
II	15/18	21/30	1.875 (0.955 to 3.681)	0.068
IIIA	23/29	22/31	1.027 (0.567 to 1.860)	0.930
IIIB	13/14	13/14	0.800 (0.359 to 1.783)	0.585
IV	18/18	8/11	1.649 (0.706 to 3.850)	0.248
Gross type (Borrmann)				
I	2/3	2/2	1.405 (0.125 to 15.838)	0.783
II	18/20	7/12	1.908 (0.790 to 4.609)	0.151
III	50/59	46/66	1.536 (1.028 to 2.296)	0.036^c^
IV	16/17	23/29	1.623 (0.847 to 3.111)	0.145
Type of gastrectomy				
Total	53/60	50/68	1.317 (0.893 to 1.942)	0.165
Sub-total	34/40	33/46	1.700 (1.032 to 2.803)	0.037^c^

*Abbreviations*: *G + S* gastrectomy plus splenectomy, *G-A* gastrectomy alone, *HR* hazard ratio, *TNM* tumor, node, metastasis.

^a^HR >1 indicates that G-A was superior, whereas HR <1 indicates that G + S was superior.

^b^The *P*-values are for HRs for deaths in each group (G + S or GA), with 95% CI.

^c^Considered significantly better for G + S.

## Discussion

The spleen is an organ that protects the host against infection and perhaps also against tumor micrometastases, and knowledge about its immunological functions has increased markedly in recent years. Although lymph nodes in the hiluma of the spleen may be affected by gastric tumors, the spleen itself is seldom affected. Our retrospective comparison showed that OS was significantly higher and complication rates significantly lower in patients undergoing G-A than in those undergoing G + S for curative resection of gastric cancer.

More extended surgery is associated with increased risks of operative morbidity and mortality. We found that removal of the spleen during the course of resection for gastric carcinoma significantly increased operative morbidity rates compared with spleen preservation (24% versus 10%, *P* < 0.05). The increased morbidity after splenectomy may due to the higher incidence of infectious complications after gastrectomy. Mortality rates in western countries for patients undergoing gastrectomy often exceed 5% and may be as high as 16% [2]. Thus, G + S should not be considered the standard curative surgical procedure in patients with gastric cancer unless the addition of splenectomy is found to significantly benefit patients by reducing operative morbidity and mortality rates or improving long-term survival. In the current study, no evidence was found that removal of the spleen led to any increase in 5-year OS rate after potentially curative resection. We found that the 5-year OS rates were 28.8% in patients undergoing G + S and 33.8% in patients undergoing G-A, similar to previous findings [4-7]. Splenectomy was also shown to negatively influence survival, and in addition, increased length of hospital stay and probability of death [8-10].

In addition to the extent of the surgery, the operative skill and experience of the surgeon(s) and the workload of cases are also important factors for survival rates [11,12]. Many studies have reported a relationship between the number of cases treated in a hospital and the outcomes of cancer treatment [12-17]. Moreover, the uniformity of treatment is also important. Our study was carried out in a hospital that performs a high volume of dissections for gastric cancer, with low morbidity and mortality rates. All participating surgeons were from the same department, which minimizes the variation in individual operating skill and management, and balances the comparisons between the two groups without bias from the skill of individual surgeons.

Because G-A is associated with lower mortality and adequate PS when performed in selected institutions with sufficient surgical experience and good postoperative management, we recommend that most patients with curable gastric cancer should undergo total or sub-total gastrectomy combined with radical lymphadenectomy, a type of surgery compatible with the preservation of the spleen.

## Conclusions

Splenectomy does not benefit patients undergoing radical resections for gastric cancer, and should not be performed.

## Competing interests

The authors declare that they have no competing interests.

## Authors’ contributions

HZ and H-MX had the original idea and designed the study. YR and C-GL were the participating surgeons, and they reviewed the surgical reports and contributed follow-up data. D-YP performed the statistical analyses, analyzed the data, and edited the paper. C-GL supervised the progress of the trial and edited the paper. All authors read and approved the final manuscript.
